# Single phase 3D phononic band gap material

**DOI:** 10.1038/s41598-017-04235-1

**Published:** 2017-06-19

**Authors:** Franziska Warmuth, Maximilian Wormser, Carolin Körner

**Affiliations:** 1 Joint Institute of Advanced Materials and Processes (ZMP) Fürth, Friedrich-Alexander University of Erlangen-Nuremberg, Dr.-Mack-Str. 81, 90762 Fürth, Germany; 2 Chair of Metals Science and Technology (WTM), Friedrich-Alexander University of Erlangen-Nuremberg, Martensstr. 5, 91058 Erlangen, Germany

## Abstract

Phononic band gap materials are capable of prohibiting the propagation of mechanical waves in certain frequency ranges. Band gaps are produced by combining different phases with different properties within one material. In this paper, we present a novel cellular material consisting of only one phase with a phononic band gap. Different phases are modelled by lattice structure design based on eigenmode analysis. Test samples are built from a titanium alloy using selective electron beam melting. For the first time, the predicted phononic band gaps via FEM simulation are experimentally verified. In addition, it is shown how the position and extension of the band gaps can be tuned by utilizing knowledge-based design.

## Introduction

Materials with complete phononic band gaps show frequency intervals in which the propagation of mechanical waves is not possible for any direction^1^. A lot of studies have been dedicated to phononic crystal systems with periodic variation of density and large mismatches in wave speed periodically modulated on a length scale comparable to the desired wavelength based on multi-phase systems^1–4^. We present a novel approach of designing the unit cell of a single phase three-dimensional cellular structure leading to complete and tunable phononic band gaps. Additive manufacturing is used to fabricate samples and verify the numerical predictions. This, in turn, opens completely new ways to adjust the vibrational and damping properties of structural components.

Materials with phononic band gaps are either phononic crystals or acoustic metamaterials. Croënne *et al*.^5^ defined phononic crystals as systems in which the periodic arrangement of scatterers in a matrix is responsible for the emergence of phononic band gaps. Acoustic metamaterials, however, rely on the characteristics of the single inclusions inside the medium^5^.

Different phononic band gap formation mechanisms have been described in literature^5^. The most common one relies on Bragg scattering of the waves in phononic crystals at the periodic inclusions and their destructive interference, hence they are called Bragg gaps^6^. Furthermore, hybridization gaps are caused by coupling of the rigid-body resonances of individual inclusions as well as the propagating mode in the embedding medium and do not need a periodic arrangement of inclusions^3, 7^. Another mechanism appears only in systems in which masses are elastically bonded: in such a mechanism the resonant modes of the masses interact via the elastic bonding and passbands are generated^5^. This weak coupling (if not too strong) leads to the formation of passbands as the resonances of the single masses are coupled (compare: electron energy levels in atoms in solids). The passbands’ frequencies are higher than the individual resonances of the single beads due to the coupling^5^. The phononic band gaps then emerge as stop bands between the passbands.

Based on theoretical and numerical methods, the existence of phononic band gaps has been predicted in lattice topologies^8, 9^, undulated lattices^10^ and three-dimensional lattices^9, 11, 12^. The size and position of phononic band gaps in cellular solids can be controlled via the topology^13^ and dimension^14^ of the underlying unit cell. Apart from the chosen geometry^9, 15^, the slenderness ratio of the struts^8^ and the angle between the struts^16^ were identified as key parameters for controlling the band structure. The addition of masses^17^ or scatterers of various forms^18^ on different locations of lattice structures was also shown to be a useful method for designing and manipulating phononic band gaps.

Although there are various examples for numerically studied phononic crystals, there is a lack of experimental verifications. Sample geometries ranged from thin beams with periodically attached masses and springs^19^ to steel spheres in water^4^, undulated beams^20^ and two-dimensional polycarbonate cellular plates^21^. It was shown experimentally that the dispersion relations of two-dimensional extruded periodic cellular structures can be altered by applying tension or compression^22^. The basic experimental setup for analysing the band structure is usually as follows: a shaker is used as a source of mechanical waves while an accelerometer is used to detect the transmitted signal. Nevertheless, up to now, no example can be found in literature for a phononic crystal realized as three-dimensional one-phase cellular structure exhibiting a significant and broad phononic band gap, as demonstrated in this letter.

Eigenfrequency analysis with periodic boundary conditions of basic topologies can be used as a systematic approach to identify interesting unit cells and basic mechanisms^15^. The eigenmode shapes from this analysis display all possible unit cell shapes derived from a basic unit cell while guaranteeing that the resulting cell shapes can be arranged in a periodic lattice^9^. This approach was already used to identify auxetic unit cells and the underlying design principle^23^.

In ref. 9, the mechanism for the emergence of phononic band gaps was identified to be a specific arrangement of bent strut elements to hamper the first collective rotational eigenmode of the studied unit cells. This eigenmode was shifted to higher frequencies and thus, opens a complete band gap. A design principle was derived to generate deep and broad band gaps. Bent struts lead to low first eigenfrequencies of the struts and therefore to a deep lower boundary of the band gap. On the other hand, clamping of the nodes results in a high first collective rotational eigenfrequency, which forms the upper boundary of the band gap.

**Figure 1:**
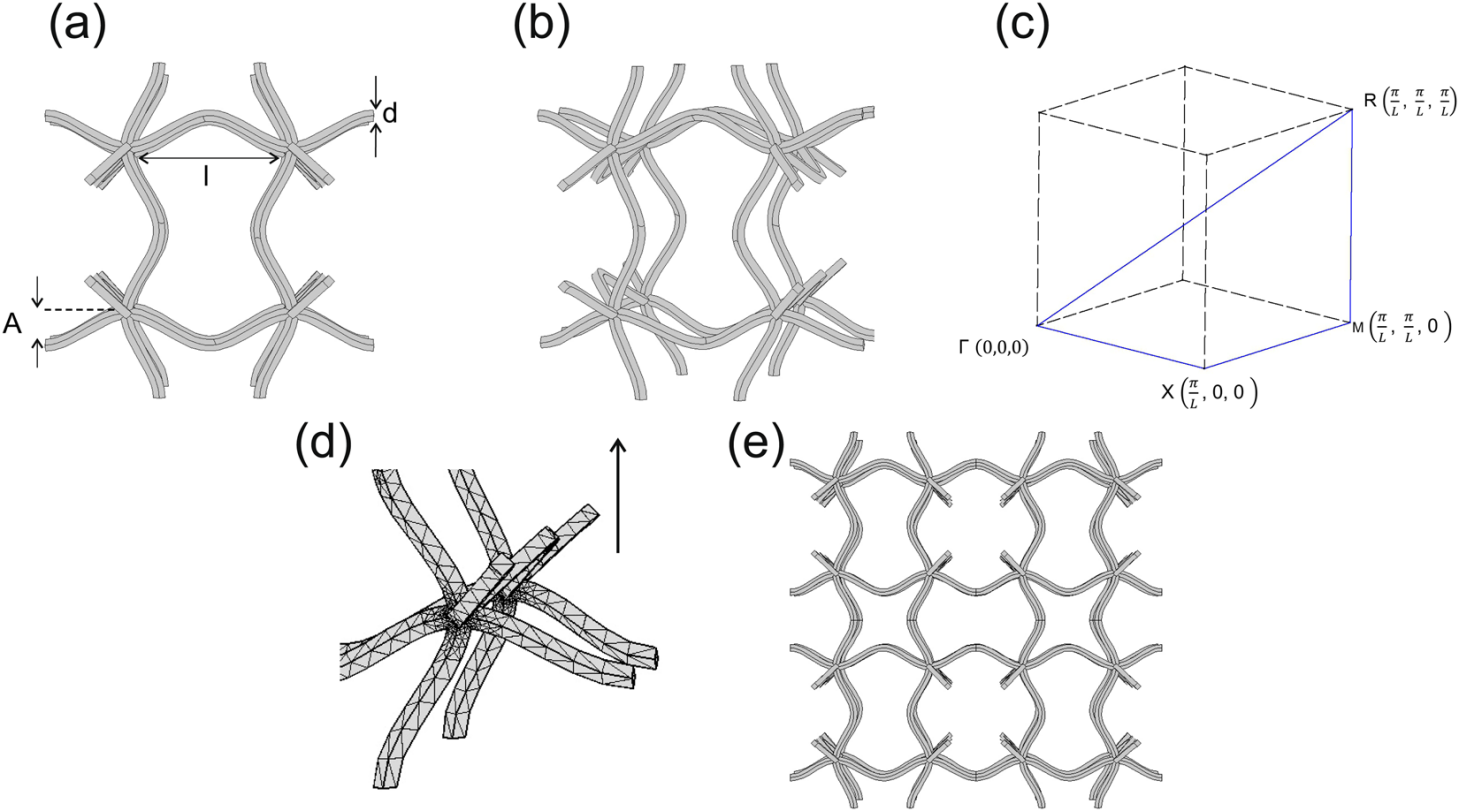
Unit cell: 22nd eigenmode of basic cubic unit cell: (**a**) View from xy-direction. (**b**) Slightly rotated view. (**c**) Path and corresponding coordinates in $\overrightarrow{k}$-space, (**d**) Meshed part of the unit cell, (**e**) Corresponding periodic lattice.

In this work, for the first time, experimental evidence of broad and complete band gaps in a single phase cellular structure is reported. The gaps emerge by means of structure-borne sound and without a matrix medium. Therefore, the gap is not a result of different elastic constants as usually present in multi-phase phononic crystals^3, 6^. Instead, the presented geometry is designed to emulate the behaviour of a dual phase material in which soft struts act as an effective medium connecting the relatively stiff nodal points. The underlying unit cell is depicted in Fig. 1, which is an eigenmode of a cubic cell where the nodes rotate in a collective way. This eigenmode can be approximated by sinusoidal functions with extremal points meeting in the nodal points. The rotation of the nodal points is hampered due to struts bending in different directions. The unit cell is further characterised by the nodal distance *l*, the strut thickness *d* and the amplitude *A* of the strut.

## Results and Discussion

For the fabrication of these cellular structures additive manufacturing is an ideal technique due to its capability to fabricate complex geometrical components. Functional parts might be added to the structure as well. Here, Selective Electron Beam Melting (SEBM) is used, which is a powder-bed based generative manufacturing technique. The powder bed is selectively molten according to sliced CAD data (see also ref. 24). Due to their high geometric freedom, both SEBM and SLM (Selective Laser Melting) have been used to build metallic lattice materials for structural applications^25^ and/or to demonstrate novel properties such as the auxetic effect^24^.

**Figure 2:**
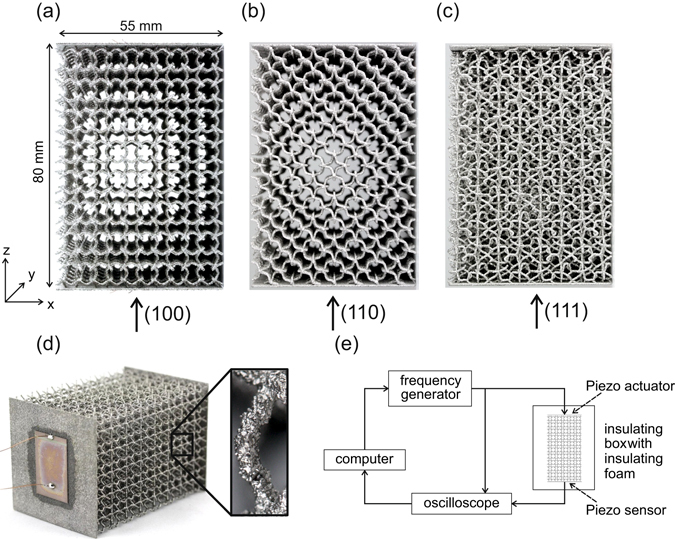
Pictures of SEBM samples made from Ti-6Al-4V powder and consisting of 8 unit cells in z-direction bordered with thin walls at each end and experimental setup. (**a**) Side view of a sample with transmission direction (100), (**b**) (110) and (**c**) (111). (**d**) Scenographic view of a (100) sample and close-up view of a strut. Piezoelectric elements are glued onto the walls. (**e**) Schematic of the experimental setup.

The (100) direction sample geometry used for band gap analysis is depicted in Fig. 2a,d. The SEBM process allows to build these structures without supports. The minimum strut thickness is determined by the electron beam diameter. As SEBM is a powder based process, the side and bottom areas of the struts are in contact with the surrounding powder bed. This leads to partially or completely unmelted powder particles at the surface and a process inherent roughness of SEBM struts^24^, as can be seen in the zoomed view in Fig. 2d. Thus, the mechanically active strut diameter will be lower than the mean strut diameter^26^.

For experimental verification, samples consisting of 5.5 × 5.5 × 8 unit cells with two thin compact side walls (0.7 mm) were manufactured from Ti-6Al-4V powder. Two piezoelectric elements were glued on the walls where the struts of the cellular structure were embedded (see Fig. 2d). The thin walls provided a suitable substrate for mounting the piezoelectric elements. The overall setup is depicted in Fig. 2e. A frequency generator is used to send a sinusoidal potential at a constant amplitude to the first piezoelectric element. The piezo actuator excites vibrations in the structure that propagate to the other side of the sample where the incoming vibrations are converted to an electric signal via the piezo sensor element. The incoming and outgoing signals are recorded. The ratio of the maximum value of the incoming and outgoing signal amplitudes is determined as a measure for the transmission. The transmission direction for all samples is along the z-axis of the sample.

In order to verify the existence of a complete phononic band gap, additional samples with two different transmission directions were used. Figure 2b,c shows samples with transmission along the crystallographic directions (110) and (111). These two crystallographic directions along with the regular (100) direction visible in Fig. 2a form the main directions of the unit cell on which a band gap has to exist to indicate a complete phononic band gap.

**Figure 3:**
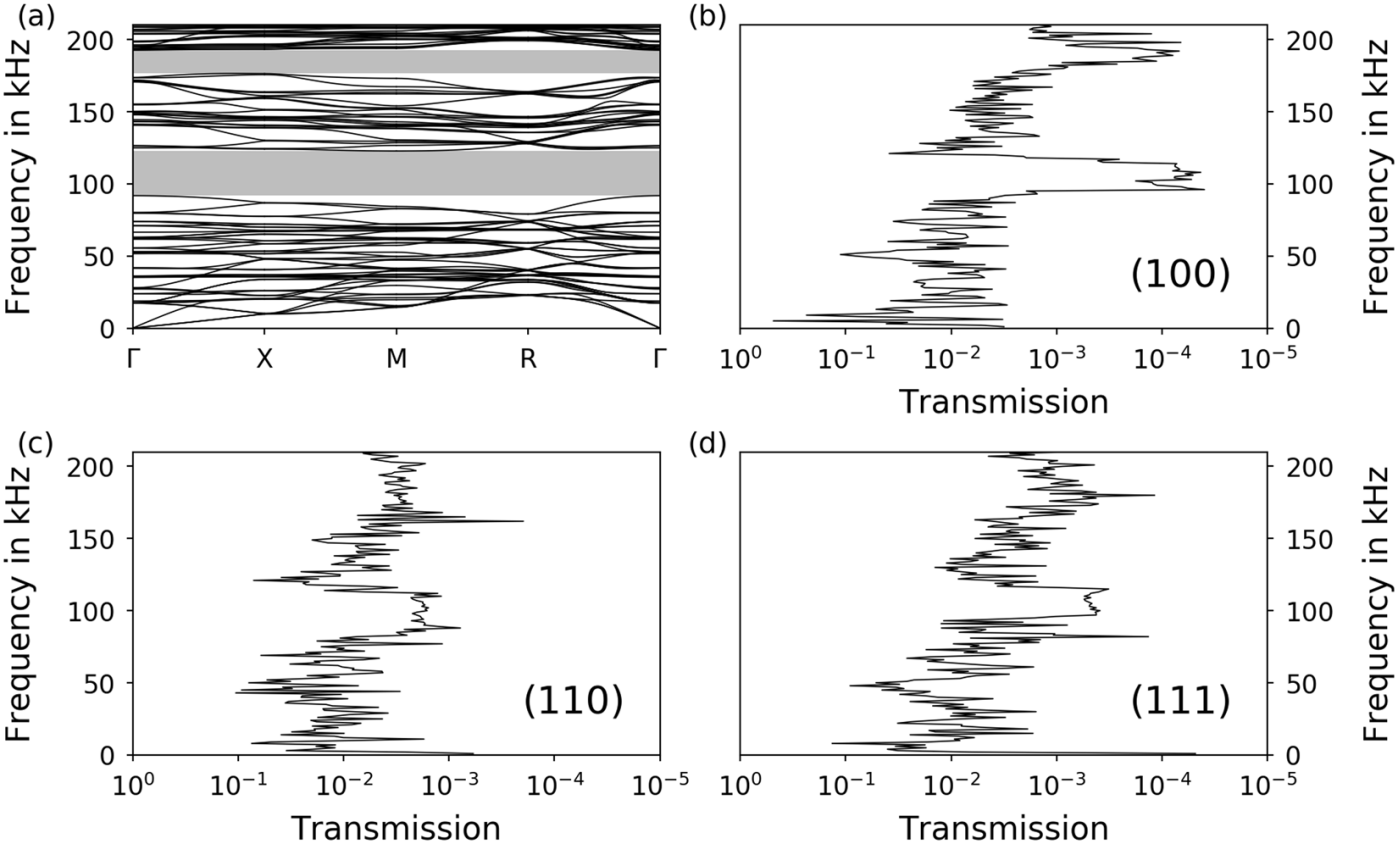
Comparison of numerical dispersion relation and experimental transmission results along different crystallographic directions (samples with l = 5.0 mm and A = 1.0 mm). (**a**) Numerical dispersion relation (d = 0.52 mm) with complete band gaps indicated in grey. (**b**) Experimental transmission spectrum of a sample with transmission direction (100), (**c**) (110) and (**d**) (111) with a thickness of 0.52 mm 0.08 mm.

A strut thickness of 0.52 mm was assumed for the FEM calculations of the dispersion relations of the unit cell in Fig. 1 with Bloch-Floquet boundary conditions. The corresponding dispersion relation along the path Γ–X–M–R–Γ can be seen in Fig. 3a. Discrete eigenfrequencies are displayed as lines while the frequency regions with complete band gaps are indicated by grey bars. Complete band gaps from 92 kHz to 123 kHz and from 176 kHz to 193 kHz are visible.

The experimental sonic transmission diagram for the (100) direction is depicted in Fig. 3b. The ratio of incoming and outgoing signal, the transmission, is strongly oscillating. A clear drop in the transmitted signal can be seen for frequencies in the range of approximately 100 kHz to 130 kHz and 180 kHz to 200 kHz. These frequency ranges correspond to the first and second band gap, respectively. The strut thickness of the sample in Fig. 3b is 0.52 mm ± 0.08 mm as determined by computer tomography (CT). Bearing in mind the rough surface and the consequence that the mechanically active diameter of the SEBM struts is difficult to measure exactly, the phononic band gaps in the experimental data correspond well with the first and second band gap in the dispersion relation in Fig. 3a when using the same strut thickness.

Figure 3c,d show transmission diagrams for the directions (110) and (111). The first band gaps are located at approximately the same frequency range compared to the (100) direction. This indicates the existence of a complete band gap, because all three directions show the first band gap in the same frequency range. As a consequence, the first band gaps of the (110) and (111) directions are also comparable to the numerical results. The differences in band gap depth are not quantifiable, since these two samples have been fabricated in a different process run. The changes can be attributed to small differences in the strut geometry, for example due to the orientation of the struts in the SEBM process. The shape accuracy is crucially dependent on the orientation of the struts. Therefore, deviations from the original geometry as in these samples can affect the outcome of the measurements. The same reasoning applies to the second band gap, which is not visible in the (110) and (111) directions. The cause for the vanishing of the second band gap in these samples needs to be further investigated.

The reason why this eigenmode of the cubic unit cell displays a deep and broad first band gap is attributed to the particular arrangement of the struts around the nodal point. The bent struts ease the linear translation of the nodal points towards each other which leads to a low first eigenfrequency of the struts (lower frequency of the band gap). At the same time, the nodal points are clamped since the struts are bent in different directions (see Fig. 1d). This leads to a hindering of the rotation of the nodes. As a result, the first collective rotational frequency, which is the upper frequency of the band gap, is increased and higher than the translational eigenmode (see also ref. 9). The coupling of the oscillation of the nodal points through hindering their rotation with the struts acting as weak elastic elements between the nodal points leads to a sort of hybridization gap as described by previous literature^3, 5, 27^. In contrast to descriptions of hybridization gap materials up until this point, though, the unique new feature of the present structure is that there is only a single phase in the material. The second gap develops in a similar way with the lower boundary being the second translational mode and the upper boundary the second collective rotational mode.

**Figure 4:**
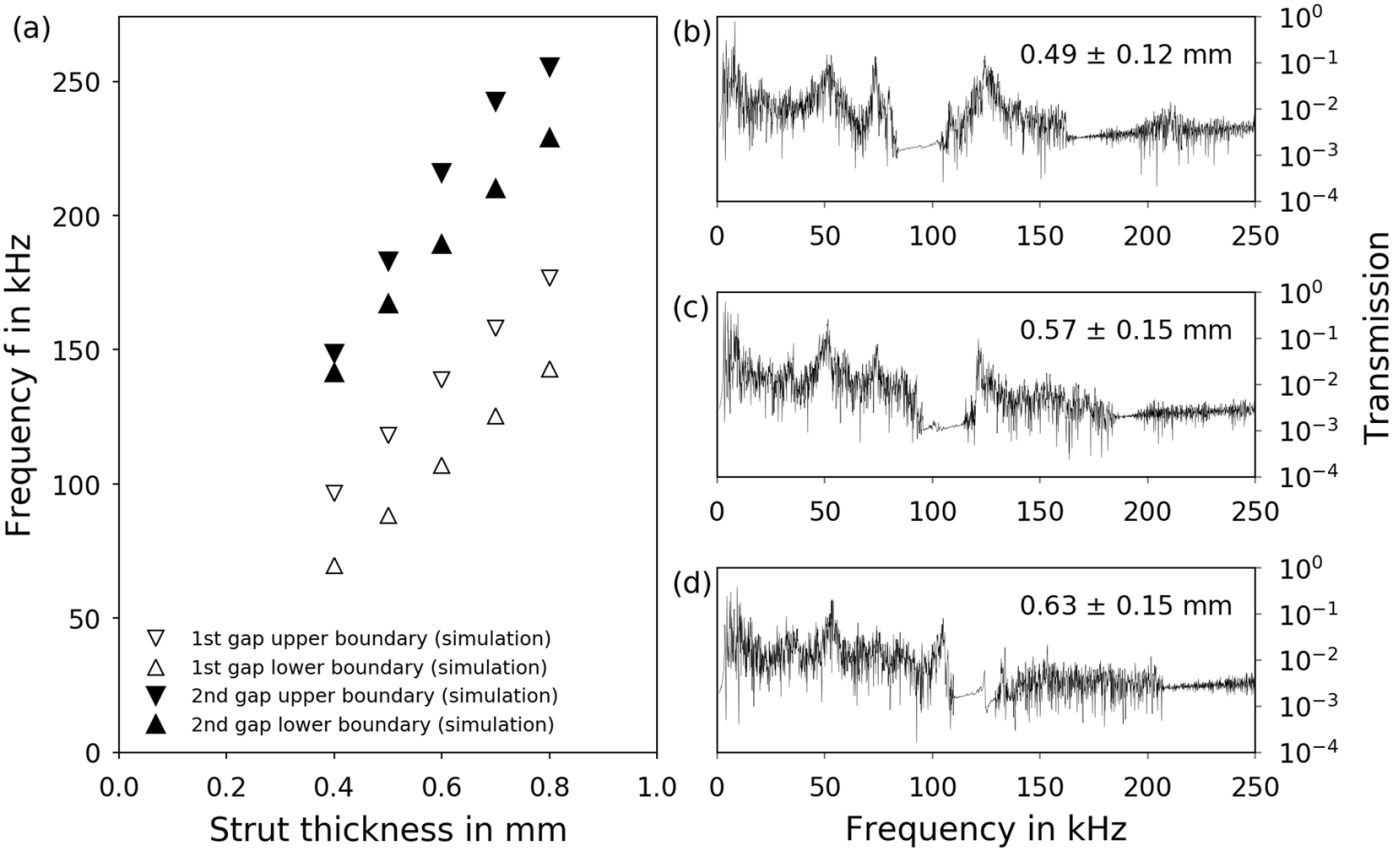
Size and position of the first and second phononic band gap in dependency of the strut thickness. (**a**) Numerical results (l = 5.0 mm, A = 1.0 mm). Experimentally obtained transmission spectra for (100) direction samples built with a line energy *E*_*L*_ of (**b**) 0.4 J/mm, (**c**) 0.6 J/mm and (**d**) 0.8 J/mm with corresponding strut thickness as measured in CT.

The lower and upper frequency of the band gap can be shifted by varying the strut thickness. Numerical results for the lower and upper frequencies of the first band gap in dependency of the strut thickness are shown in Fig. 4a. The thicker the struts, the stiffer the structure and the more difficult the excitation of the natural resonances gets. This finding was also observed for two-dimensional structures^9^. The band gap position is shifting towards higher frequencies with increasing strut thickness. For the first band gap, the lower as well as the upper boundary shift almost linearly. While the lower boundary of the second band gap behaves similarly, the upper boundary is closer to the lower boundary with thinner struts and widens the band gap with thicker struts.

Experimentally, different strut thicknesses were realized by varying line energies during manufacturing. The higher the line energy, the thicker the strut. The mean strut thickness was determined by CT measurements. The roughness of the struts due to the sticking of unmelted powder particles is reflected in the standard deviation of the determined strut thickness. The corresponding transmission spectra and thicknesses of the struts of three (100) direction samples are displayed in Fig. 4b–d. It is clearly visible how the upper boundary of the first band gap is shifted from approximately 100 kHz to 125 kHz with increasing strut thickness. Its lower boundary is shifted from approximately 80 kHz to 110 kHz with increasing strut thickness. Due to the noise in the experimental setup, the upper end of the second band gap is not pronounced. However, the lower end is clearly shifted from around 160 kHz to 210 kHz with increasing strut thickness.

Similarly to the results described in Fig. 3, the numerical and experimental results in Fig. 4 correspond in terms of band gap position and width. It can be seen that by comparing the sample with a strut thickness of 0.49 ± 0.12 mm with the numerical results of a cell with 0.5 mm struts both band gaps are situated around 100 kHz. The same comparison can be made for the other two experimental results compared to the numerical results for a strut thickness of 0.6 mm. Given the error range of the strut thicknesses in the samples due to their roughness, the experimental band gaps are very close to the numerical predictions.

The band gap frequency shift with increasing strut thickness can also be estimated by looking at the eigenfrequency equation of a strut. According to ref. 15, the first eigenfrequency of a strut depends on the length *l* and thickness *d* made of a material with Young’s modulus *E* and density *ρ* as follows

1 $$\omega \sim \sqrt{\frac{E}{\rho }}\cdot \frac{d}{{l}^{2}}.$$

This relation shows that the eigenfrequency of a strut is proportional to its thickness. The difference in strut thickness of the samples in Fig. 4b,d is approximately 25% (calculated as increase from the lower thickness) which corresponds very well to the shift of the lower band gap boundaries from 80 kHz to 110 kHz and from 160 kHz to 210 kHz for the first and second gap, respectively.

The presence of complete phononic band gaps in cellular structures was predicted by using dispersion relations obtained via FEM and Bloch-Floquet boundary conditions. Using Selective Electron Beam Melting, cellular structures consisting of the studied unit cell were manufactured. A suitable experimental setup utilizing piezoelectric elements as actuator and sensor was built. For the first time, a complete broad band gap was measured in a single phase three-dimensional sample. The location of the band gaps can be designed by varying the strut thickness. Numerical and experimental results are in good agreement.

## Methods

### Numerical eigenfrequency analysis

Starting from a basic cubic unit cell, an eigenfrequency analysis with periodic boundary conditions was conducted by using FEM software Comsol Multiphysics 5.0. 100 eigenmodes were calculated. For the work presented here, the collective rotational eigenmode with fixed rotational centers (see also ref. 9) was chosen.

To apply Bloch-Floquet boundary conditions, the eigenmode was re-drawn using a CAD software (Autodesk Inventor) as otherwise the mesh elements were too distorted to apply Bloch-Floquet boundary conditions.

The dispersion relation was calculated by determining the first 250 eigenfrequencies of the unit cell at 25 points along each part of the path Γ–X–M–R–Γ in $\overrightarrow{k}$-space. The mesh consisted of tetragonal elements with a size between 0.18 and 1.0 mm. The used material parameters correspond to titanium (Young’s modulus E = 105 GPa, density *ρ* = 4940 kg/m^3^, Poisson’s ratio *ν* = 0.33). A comprehensive introduction to the combination of the FEM method and Bloch-Floquet boundary conditions is given in ref. 28.

### Fabrication of the samples

An Arcam AB Q10 machine was used to build the structures by Selective Electron Beam Melting (SEBM). Ti-6Al-4V powder with a particle size between 45 and 105 μm was used. At an acceleration voltage *U* of the electrons of 60 kV a beam current *I* of 3 mA at varying speeds was applied. The build parameters are presented in Table 1. The line energy *E*_*L*_ can be calculated as

2 $${E}_{L}=\frac{U\ast I}{v}.$$

The measured samples consisted of 5.5 × 5.5 × 8 unit cells. On two opposing ends, the sample ends with two thin walls that are directly connected and built as part of the sample in the process. The unit cell had a nodal distance *l* of 5.0 mm, an amplitude *A* of 1.0 mm and a strut thickness *d* of 0.25 mm in the CAD file. Two piezo electric elements (20 mm * 30 mm * 0.2 mm, PI Ceramic) were glued on the walls directly connected to the structure using a thin layer of an instant adhesive based on acrylate. In a last step, the two electrodes of the piezo-elements were contacted using tin solder and thin copper wires.

**Table 1:** Beam parameters for building the samples: voltage *U*, electron beam current *I*, electron beam speed *v*, line energy *E*_*L*_ and resulting strut thickness *d*.

*U* [kV]	*I* [mA]	*v* [mm/s]	*E*_*L*_ [J/mm]	*d* [mm]
60	3	450	0.40	0.49 ± 0.12
60	3	300	0.60	0.57 ± 0.15
60	3	225	0.80	0.63 ± 0.15

The strut thickness was measured using computer tomography (MicroCT 40, Scanco Medical AG, Switzerland) at an acceleration voltage of 50 kV, a current of 160 μA and a voxel size of 15 μm. The threshold for the evaluation was calibrated with a sample of known thickness. The strut thickness was measured on single unit cells that were built with the same parameters as the measured samples.

### Sonic Transmission Measurement

During the measurement, the sample was situated on insulating polymer foam in an insulating box to prevent external interferences. The first piezo element acted as an actuator while the second piezo element was used as a sensor. By using a frequency generator, sinusoidal potential signals with a constant amplitude of 10 V with frequencies between 0 and 250 kHz were sent to the first piezo element. An oscilloscope was used to record both, the incoming and the outgoing signal at the two piezo elements. The transmission was characterized by the quotient of the maximum measured amplitudes of sensor signal to actuator signal.
